# Correction to: 7.R. Health promotion interventions

**DOI:** 10.1093/eurpub/ckac185

**Published:** 2021-12-31

This is a correction to: 7.R. Health promotion interventions, *European Journal of Public Health*, Volume 32, Issue Supplement_3, October 2022, https://doi.org/10.1093/eurpub/ckac130.141

Upon first publication this abstract was incorrectly marked as having been withdrawn. This notice is to notify readers that the abstract should not have been withdrawn and has now been replaced.

